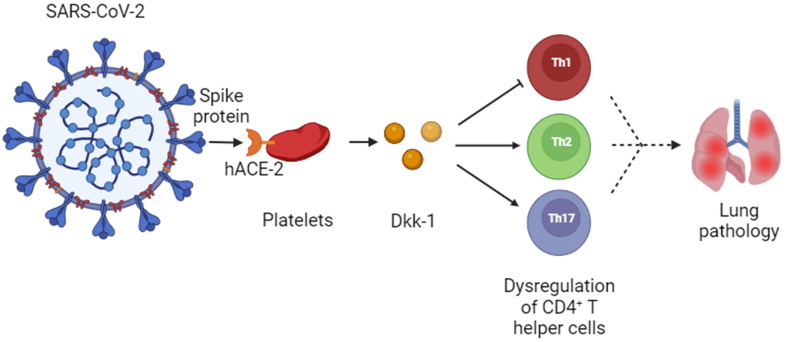# SARS-CoV-2 triggers Dickkopf-1 (Dkk-1) modulation of T helper cells and lung pathology in mice

**DOI:** 10.1016/j.gendis.2023.101167

**Published:** 2023-11-15

**Authors:** Yifan Wu, Freedom M. Green, Stephen A. Shaw, Lauren J. Bonilla, Shannon E. Ronca, Maria Elena Bottazzi, Farrah Kheradmand, Jill E. Weatherhead

**Affiliations:** Department of Pathology & Immunology, One Baylor Plaza, Houston, TX 77030, USA; Department of Pediatrics, One Baylor Plaza, Houston, TX 77030, USA; Department of Medicine, One Baylor Plaza, Houston, TX 77030, USA; Department of Pediatrics, One Baylor Plaza, Houston, TX 77030, USA; Department of Pediatrics, One Baylor Plaza, Houston, TX 77030, USA; National School of Tropical Medicine, One Baylor Plaza, Houston, TX 77030, USA; Molecular Virology & Microbiology, One Baylor Plaza, Houston, TX 77030, USA; Department of Pediatrics, One Baylor Plaza, Houston, TX 77030, USA; National School of Tropical Medicine, One Baylor Plaza, Houston, TX 77030, USA; Department of Pathology & Immunology, One Baylor Plaza, Houston, TX 77030, USA; Department of Medicine, One Baylor Plaza, Houston, TX 77030, USA; Biology of Inflammation Center, Baylor College of Medicine, One Baylor Plaza, Houston, TX 77030, USA; Michael E. DeBakey VA Center for Translational Research on Inflammatory Diseases, Houston, TX 77030, USA; Department of Pediatrics, One Baylor Plaza, Houston, TX 77030, USA; Department of Medicine, One Baylor Plaza, Houston, TX 77030, USA; National School of Tropical Medicine, One Baylor Plaza, Houston, TX 77030, USA

Severe acute respiratory syndrome coronavirus 2 (SARS-CoV-2) is the etiologic agent of the coronavirus disease 19 (COVID-19) pandemic. In viral infections like SARS-CoV-2, a robust anti-viral type 1 immune response, including up-regulation of type 1 CD4^+^ T helper cells (Th1), is required for effective viral clearance while minimizing end-organ pathology and collateral tissue damage. However, in severe SARS-CoV-2 infection, T cell dysregulation can occur, leading to reduced Th1 and elevated Th2 and Th17 cells, exaggerated T cell-mediated cytokine release, and extensive systemic inflammation and tissue damage known clinically as severe COVID-19.^1^ Despite the global impact of severe COVID-19, the molecular mechanisms of T cell dysregulation during SARS-CoV-2 infection remain largely unknown. We previously discovered that platelet-derived Dickkopf-1 (Dkk-1), a Wnt signaling inhibitor, is a critical regulatory molecule in pulmonary T cell immune responses during pulmonary infections and induces Th2 and Th17 cell differentiation and activation.^2^ Additionally, the Dkk-1 signaling pathway is a potential biomarker for COVID-19-induced lung injury.^3^ We hypothesize that platelet-derived Dkk-1 is a key regulator in SARS-CoV-2-associated T cell dysregulation leading to severe COVID-19.

We used plasma samples from patients hospitalized with COVID-19 enrolled in the immunophenotyping assessment in a COVID-19 cohort (IMPACC).^4^ Plasma specimens were collected as part of the IMPACC study and permission to use the specimens for this study was obtained from the IMPACC steering committee.^4^ Hospitalized patients with acute COVID-19 had significantly elevated plasma Dkk-1 by ELISA compared with healthy controls. Furthermore, plasma Dkk-1 levels reduced towards normal at the time of clinical recovery, indicating Dkk-1 is up-regulated in patients with active SARS-CoV-2 infection (Fig. 1A). We subsequently verified that ancestral SARS-CoV-2 spike (S) protein activates human platelets through angiotensin-converting enzyme 2 receptor (ACE-2)^5^ by measuring CD62P expression using flow cytometry *in vitro* (Fig. S1A–C). Furthermore, treatment of human platelets *in vitro* with escalating doses of S protein led to a dose-dependent release of Dkk-1 (Fig. 1B) which was inhibited by treatment with a human angiotensin-converting enzyme 2 receptor (hACE-2) antagonist (Fig. 1C). This indicates the role of S protein and platelet hACE-2 interaction in the release of platelet-derived Dkk-1. Using a hACE2-transgenic (tg) mouse model, mice were challenged intranasally with S protein, and Dkk-1 concentrations were measured by ELISA. Similar to the *in vitro* findings, S protein-challenged hACE2-tg mice had elevated Dkk-1 in both plasma and isolated platelets collected from plasma (Fig. S2A). Similarly, we verified that intranasal challenge with SARS-CoV-2 delta variant S protein (SΔ) in hACE2-tg mice led to the elevation of Dkk-1 as well (Fig. S2B).

**Figure 1:**
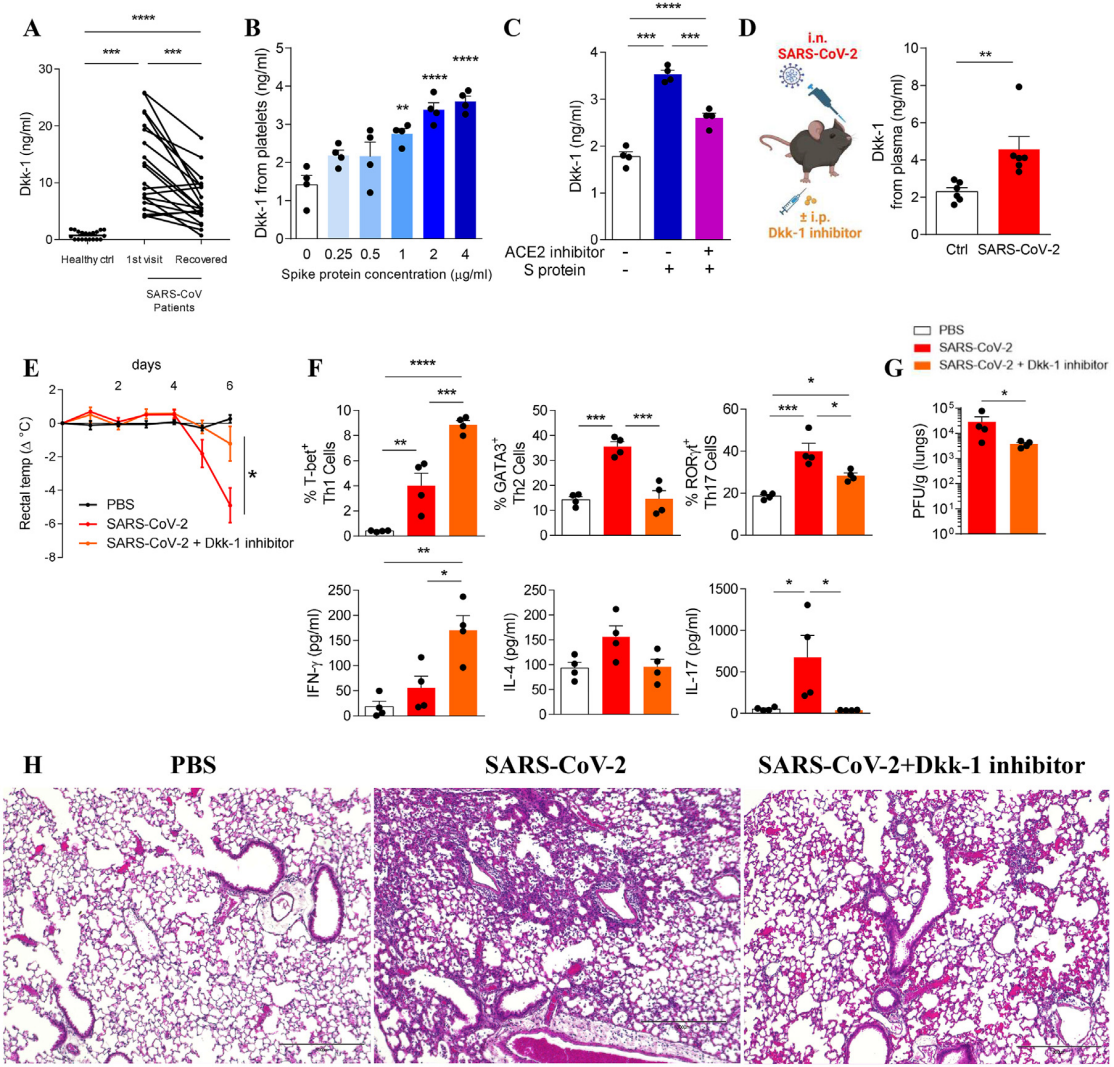
SARS-CoV-2 spike protein induces the release of platelet-derived Dkk-1 impacting cellular immune differentiation and end-organ disease during infection. **(A)** Plasma Dkk-1 levels in healthy controls (ctrl), patients with acute COVID-19, and at clinical recovery. Elevated plasma Dkk-1 was detected with active COVID-19 which decreased at time of clinical recovery. Human-derived platelets were treated *in vitro***(B)** with escalating concentration of S protein (2 μg/mL) or **(C)** with S protein (2 μg/mL) with or without hACE2 inhibitor, and Dkk-1 release was quantified by ELISA. Dkk-1 levels were elevated in human platelets following S protein challenge in a dose dependent manner. However, Dkk-1 levels were decreased in platelets challenged with S protein when they were treated with a hACE-2 antagonist. **(D)** hACE-2 transgenic mice were intranasally challenged with SARS-CoV-2 and Dkk-1 was quantified in the plasma from. Dkk-1 was up-regulated in the plasma of mice challenged with SARS-CoV-2. Subsequently, hACE-2tg mice were intranasally challenged with SARS-CoV-2, with or without Dkk-1 inhibitor and **(E)** rectal temperature changes in mice post infection were measured. Significant reduction of rectal temperature was found post-infection in SARS-CoV-2 infected mice but not in SARS-CoV-2 infected mice treated with a Dkk-1 inhibitor. **(F)** The percentage of Th1 (T-bet^+^), Th2 (GATA-3^+^), and Th17 (RORγt^+^) cells from CD45^+^, CD3^+^, CD4^+^ T cells, as well as hallmark cytokines, IFN-γ, IL-4, and IL-17 were measured from whole lung on day 6 post-infection using flow cytometry and ELISA. SARS-CoV-2 infected mice developed dysregulated T helper cell responses in the lungs including elevated Th1 and Th17 and reduced Th1 cells. However, Dkk-1 antagonism using a Dkk-1 inhibitor restored T helper cell distribution towards type1 immune response. **(G)** Viral titers from lung homogenate were measured using plaque assay in SARS-CoV-2 infected mice. Treatment with a Dkk-1 inhibitor reduced viral titers in the lungs. **(H)** Hematoxylin and eosin staining of lung sections from mice were analyzed and demonstrated that treatment of SARS-CoV-2 infected mice with a Dkk-1 inhibitor led to reduced immune cell infiltration and lung pathology. *n* = 4 mice, *n* = 20 patients; mean ± standard error of the mean; ^∗^*P* < 0.05, ^∗∗^*P* < 0.01, ^∗∗∗^*P* < 0.001, and ^∗∗∗∗^*P* < 0.0001; one-way ANOVA followed by (A, B) Dunnett's test and (C, E, F) Tukey's test for multiple comparisons; paired (A) and unpaired (D, G) student's *t*-test. Specifically, Figure 1A was analyzed using both one-way ANOVA between all groups and paired student's *t*-test between COVID-19 patients, which both showed at least ^∗∗∗^*P* < 0.001. Magnification: 100 × . Scale bar: 300 μm. Illustrative figures were generated at biorenders.com. The data were representative of at least two independent experiments.

To further evaluate the role of Dkk-1 in severe COVID-19 phenotypes, we infected hACE2-tg mice with ancestral SARS-CoV-2 and measured Dkk-1 in plasma. On day 6 post-infection, hACE2-tg mice infected with SARS-CoV-2 had significant concentrations of plasma Dkk-1 compared with naïve controls (Fig. 1D). We subsequently infected hACE2-tg mice with SARS-CoV-2 in the presence and absence of a Dkk-1 inhibitor that antagonizes the Dkk-1 signaling pathway.^2^ While treatment with a Dkk-1 inhibitor did not significantly prevent weight loss (Fig. S3A), treatment with a Dkk-1 inhibitor did prevent significant temperature loss which is classically observed in SARS-CoV-2 infected mice (Fig. 1E). Notably, treatment with a Dkk-1 inhibitor up-regulated Th1 and suppressed Th2 and Th17 cells measured by flow cytometry in the lungs of SARS-CoV-2 infected mice. These findings corroborated with cytokine analyses including IFN-γ, IL-4, and IL-17 by ELISA (Fig. 1F; Fig. S3B, C). Furthermore, treatment with a Dkk-1 inhibitor lowered SARS-CoV-2 viral load in the lungs of infected mice measured by viral plaque assay (Fig. 1G). The lower viral load in mice treated with a Dkk-1 inhibitor suggests that Dkk-1 inhibition restores anti-viral immune function leading to enhanced SARS-CoV-2 clearance. Finally, hematoxylin and eosin staining on lung sections revealed reduced immune cell infiltration in the lungs of infected mice treated with a Dkk-1 inhibitor when compared with their untreated controls (Fig. 1H). Thus, blocking Dkk-1 signaling through administration of a Dkk-1 inhibitor reduces collateral lung damage secondary to SARS-CoV-2 infection.

Together, these data demonstrate that platelet-derived Dkk-1 is a key regulatory molecule for T cell responses during SARS-CoV-2 infection. In other pulmonary pathogen models, like *Candida albicans*, Dkk-1 has been shown to play a key role in pulmonary mucosal immunity by stimulating type 2 and type 17 immune responses and inhibiting type 1 immune response in the lungs.^2^ However, whether Dkk-1 has a direct effect on T cell differentiation and/or function, and the mechanism through which Dkk-1 signals, remains unknown. As a Wnt/beta-catenin pathway antagonist, Dkk-1 may engage additional immune pathways that are critical for T helper cell activation and proliferation. Future studies will characterize the molecular mechanisms of Dkk-1 modulation of T cell responses in the lungs.

Our previous study discovered that resident, pulmonary megakaryocytes and platelets are indispensable first responders to pulmonary pathogens.^2^ In this process, platelets release the majority of Dkk-1 which acts as a critical regulator for T cell responses and drives pulmonary pathology during pulmonary infections. Most importantly, Dkk-1 has been identified as a biomarker for severe COVID-19,^3^ with Dkk-1 plasma concentrations following clinical recovery (Fig. 1A). In this study, plasma Dkk-1 concentrations decreased during the clinical recovery phase in patients hospitalized with COVID-19 in this study but did not reach baseline levels. The decreasing but persistent Dkk-1 levels are likely due to ongoing post-viral immune activation. We anticipate that over time, the concentration of Dkk-1 would go back to baseline levels however this will be evaluated in a future longitudinal study. Additional understanding into how SARS-CoV-2 influences megakaryocytes' up-regulation of Dkk-1 will also provide global insight into innate host immune responses in the lungs, not only to SARS-CoV-2 but to all pulmonary pathogens.

Considering the rapid emergence of several coronaviruses (SARS-CoV-1, MERS-CoV, and SARS-CoV-2) over the past 20 years, novel interventions that may be useful for future coronaviruses are urgently needed. Our results have shown that inhibition of host-derived Dkk-1 during SARS-CoV-2 infections restores T cell distribution leading to enhanced SARS-CoV-2 viral clearance and reduced collateral lung damage (Fig. 1G, H). This suggests that Dkk-1 may be a therapeutic target to prevent the progression of SARS-CoV-2 infection and potentially other coronaviruses. To further evaluate the therapeutic potential of Dkk-1 in COVID-19, additional longitudinal studies are necessary. For example, investigation of the survival benefit of Dkk-1 inhibition in mice under sub-lethal doses of SARS-CoV-2 infection would provide a more in-depth evaluation of Dkk-1 inhibitor's clinical benefit. Longitudinal studies on the pattern of Dkk-1 up-regulation, as well as its correlation with type 1, type 2, or type 17 cytokines in patients with severe COVID-19 will also be valuable. In summary, Dkk-1 is potent regulator of the lung immune response during SARS-CoV-2 infection and is promising drug target for prevention of severe COVID-19.

## Ethics declaration

This study was approved by the Institutional Animal Care and Use Committee of Baylor College of Medicine and followed federal guidelines. All mice were bred and housed at the American Association for Accreditation of Laboratory Animal Care-accredited vivarium at Baylor College of Medicine under specific-pathogen-free conditions. Plasma specimens were collected as a part of the immunophenotyping assessment in a COVID-19 cohort (IMPACC) study and were permitted to be utilized in this study by the IMPACC steering committee.

## Author contributions

JW, MEB, and FK conceived, designed, and supervised the project. YW designed and performed essential experiments, analyzed data, and wrote the initial manuscript draft. FMG, SAS, and LJB performed essential experiments. SER provided essential reagents and technical advice. All authors edited the manuscript.

## Conflict of interests

The authors have declared that no conflict of interests exists.

## Figures and Tables

**figs1:**