# Supplementary material for: SARS-CoV-2 triggers Dickkopf-1 (Dkk-1) modulation of T helper cells and lung pathology in mice
Source: Genes Dis. 2023 Nov 15;11(4):101167. doi: 10.1016/j.gendis.2023.101167 (PMC10865256; doi:10.1016/j.gendis.2023.101167)
Supplement: Multimedia component 1 [file mmc1.pdf]

Supplemental Figure 1

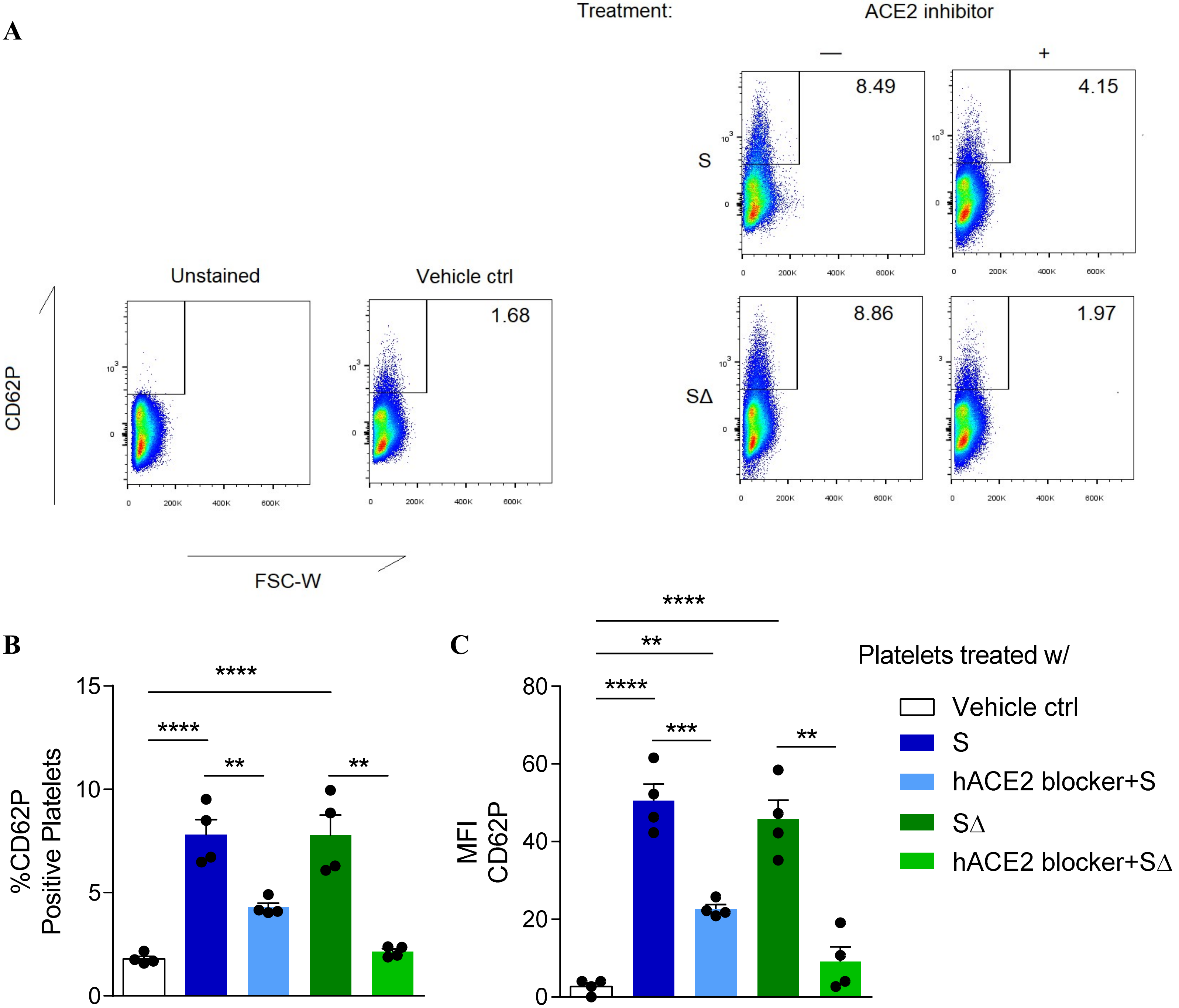

Supplemental Figure 1: Human platelets are activated by SARS-CoV-2 spike protein via ACE2. Human derived platelets are pre-treated with or without hACE2 antagonist before stimulation of spike protein (S) at 2μg/ml. **(A-C)** Platelet activation was quantified by **(B)** CD62P positive platelets and **(C)** median fluorescence intensity (MFI). (n=4, mean±S.E.M, $**p<0.01$, $***p<0.001$ and $****p<0.0001$ using one-way ANOVA followed by Tukey's test for multiple comparison)

Supplemental Figure 2

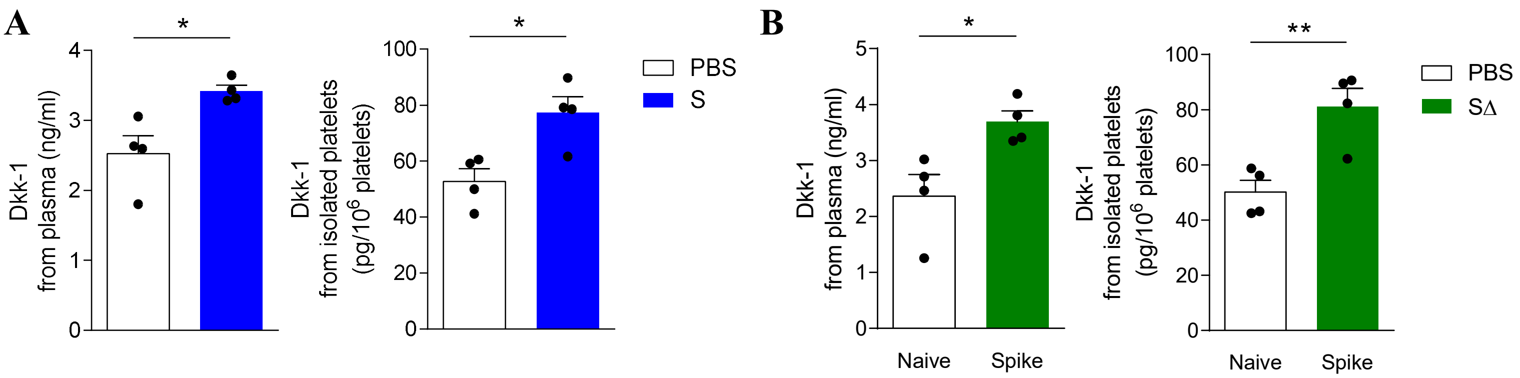

Supplemental Figure 2: Spike protein from SARS-CoV-2 triggers Dkk-1 release *in vivo*. (A-B) hACE-2 transgenic mice were intranasally challenged with recombinant (**A**) S or (**B**) SΔ, and Dkk-1 was quantified in the plasma and in platelets. (n=4, mean±S.E.M, *p<0.05, and **p<0.01 using unpaired student's t-test)

Supplemental figure 3

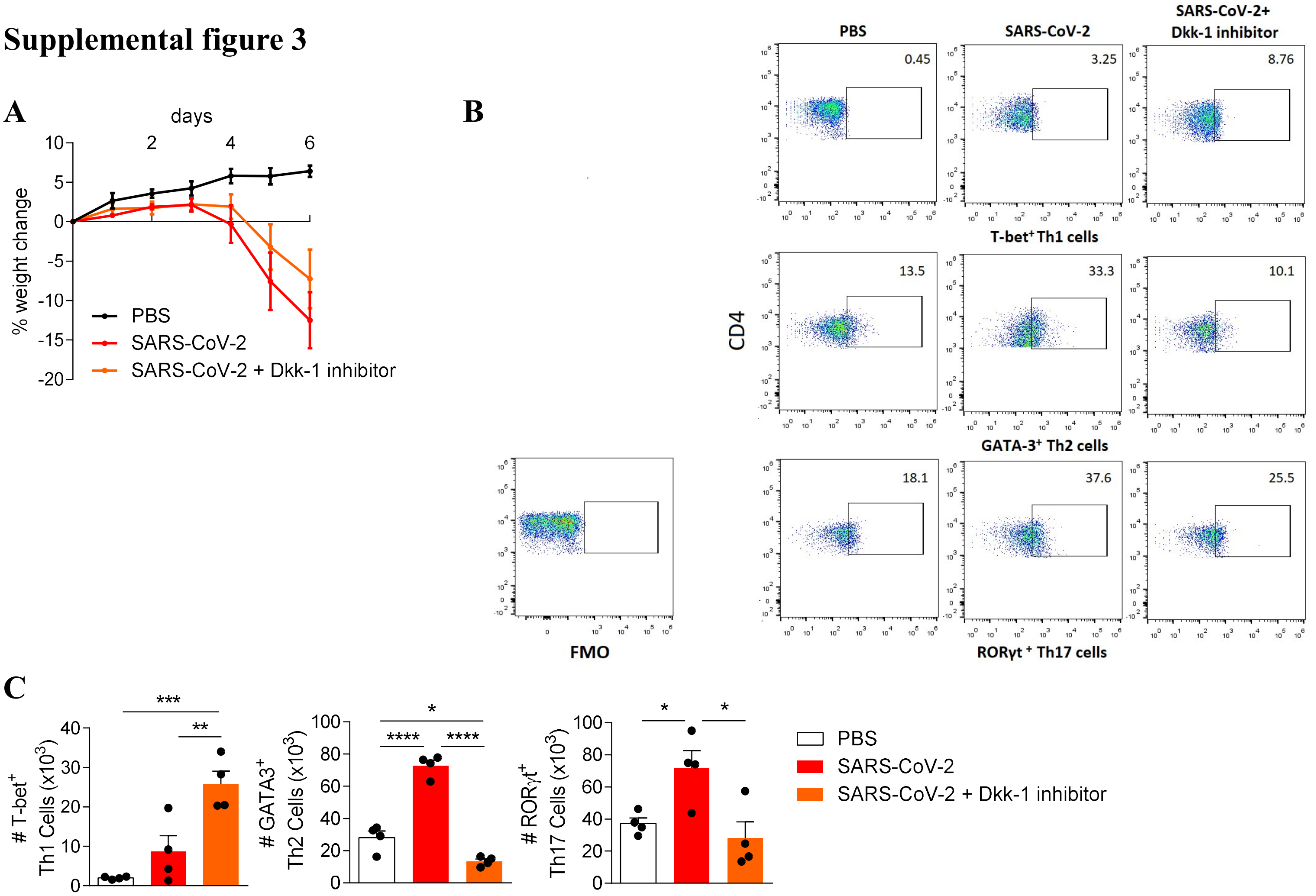

Supplemental Figure 3: Effect of Dkk-1 inhibitor on SARS-CoV-2 treated mice. hACE2 transgenic mice were challenged with SARS-CoV-2, with or without Dkk-1 inhibitor. **(A)** Percent body weight change after infection. **(B)** Representative flow plot and (**C**) total cell count gated on $CD45^+$, $CD3^+$, $CD4^+$ T cells from whole lung. (n=4, mean±S.E.M, *p<0.05, **p<0.01, ***p<0.001 and ****p<0.0001 using one-way ANOVA followed by Tukey's test for multiple comparison)

Supplemental Methods:

Methods:

Human samples: Clinical biological samples from hospitalized patients with COVID-19 have been collected as a part of the Immunophenotyping assessment in a COVID-19 cohort (Immunophenotyping assessment in a COVID-19 cohort, IMPACC) study from hospitalized COVID-19 patients at the Michael E. DeBakey Veterans Affairs Medical Center. Samples collected at the time of hospitalization and 1 year post hospital discharge were utilized. Samples from healthy donor have been collected from the Gulf Coast Blood Center, Houston, TX. 20 patient samples were included per group.

Dkk-1 release assay from human platelets: Human platelets were isolated from whole blood as platelet rich plasma through the Gulf Coast Blood Center, Houston, TX. Human platelets ($1x10^9$/mL) were pretreated with/without hACE2 blocker (MLN-4760, Millipore Sigma, Burlington, MA) for 1 h at 37°C, and then incubated overnight with escalating dose (0-4 µg/ml) of recombinant spike protein (WT: 11058-CV, Δ: 10878-CV, R&D Systems). Dkk-1 release was quantified by ELISA (DY1906, R&D systems, Minneapolis, MN).

Spike protein activation of human platelets: Human platelets in platelet rich plasma ($1x10^9$/mL) were treated with/without hACE2 blocker (MLN-4760, Millipore Sigma, Burlington, MA) for 1 h at 37°C, and then incubated with 1 µg/ml recombinant spike protein (WT: 11058-CV, Δ: 10878-CV, R&D Systems) for 1 h at 37°C. Platelet were then stained with antibodies for flow cytometry for 30 minutes at room temperature, and then centrifuged at 800xg for 5 minutes. Pellet was gently resuspended in tyrode's buffer (J67607.AP, Thermofisher scientific, Waltham MA).

Mice: 8 week-old K18-hACE2 male and female mice were purchased from Jackson Laboratories (cat: 034860). Mice were challenged with recombinant spike protein (WT: 11058-CV, Δ: 10878-CV, R&D Systems) at 250 µg/kg intranasally and euthanized 24 hours post challenge. Alternatively, mice were infected with ancestral SARS-CoV-2 (USA-WA1/2020, 1E5 plaque forming units), with or without treatment of Dkk-1 inhibitor (317700, Sigma Aldrich) at 40 µg/kg i.p. for 3 times every other day. Mice were euthanized 6 days post infection. All mice were bred and housed at the American Association for Accreditation of Laboratory Animal Care-accredited vivarium at Baylor College of Medicine under specific-pathogen-free conditions. All experimental protocols were approved by the Institutional Animal Care and Use Committee of Baylor

College of Medicine and followed federal guidelines.

Plasma isolation from mice: Whole blood from mice was isolated by retro-orbital puncture and anticoagulated with 10% 0.5 M EDTA and plasma was isolated by centrifugation at 1000 × g for 10 min at 4°C and stored at -80oC until analyzed. Plasma from either mice or humans was diluted 1:10 for Dkk-1 measurement by ELISA (DY1765, R&D systems, Minneapolis, MN).

Platelet isolation from mice. Whole blood from mice was isolated by retro-orbital bleeding and anticoagulated with 10% 0.5 M EDTA. Platelet rich plasma was isolated by centrifugation at 180 × g for 10 min at room temperature, and platelets were isolated by centrifugation at 1250 × g for 10 min at room temperature. Platelets were then resuspended in Tyrodes buffer and lysed via sonication. Dkk-1 release was measurement by ELISA (DY1765, R&D systems, Minneapolis, MN).

Plaque assay: Lungs were harvested after euthanasia of infected mice, weighed, homogenized and serial diluted. Vero E6 cells (CRL-1586, ATCC) were seeded in 12 well plates to confluency and homogenate dilutions were added. Cells were inclubated for 45 minutes at 37°C. Overlay media were introduced to each well (1:1:1, 1.5% agarose, 2xMEM, complete DMEM) and incubated at 37°C for 2 days. Each well were then fixed with 10% formalin, and stained with crystal violet. Plaques were counted 24 hours later after plate drying.

Preparation of lungs from mice: Lungs were cut into small pieces and incubated in digestion buffer (2mg/ml collagenase (#LS004177, Worthington), 0.04mg/ml DNAse (#10104159001, Sigma) 1, 20% FBS in HBSS) for 1 h at 37°C after which they were deaggregated by pressing through a 40 μM nylon mesh and centrifuged at 400 × g for 5 minutes at 4°C. Supernatants were discarded, and 1.5 mL of ACK (Thermofisher scientific, Waltham MA) was added and incubated for 3 min at room temperature for erythrocyte lysis. ACK was then neutralized with 7.5 mL of complete RPMI-1640 (Corning, NY), with 10% FBS and 1% Pen Strep, Gibco, Waltham MA). The resulting leukocyte preparations were centrifuged and prepared for flow cytometry analysis or ELISA.

ELISA: Standard ELISA was carried out using Duoset kit for Dkk-1, IFN-γ, IL-4, IL-17 (DY1765, DY1906, DY485, DY405, DY317, R&D systems, Minneapolis, MN).

Flow cytometry: Platelets were stained with CD62p (304910, Biolegend). Total lung cells were stained with CD45, CD3,

CD4 (103122, 100222, 100412, Biolegend, San Diego, CA). Cells were separated into 3 groups, then permeabilized and fixed using Transcription Factor Buffer Set and stained individually for T-bet, GATA3 or RORγt (562574, 561265, 560074, 562607, BD Biosciences, San Jose, CA).

Statistical analysis: Data are presented as means ± standard errors of the means. Significant differences relative to appropriate controls are expressed by P values of <0.05, as measured two tailed Student's t-test or one-way ANOVA followed by Dunnett's test or Tukey's test for multiple comparison. Data normality was confirmed using the Shapiro-Wilk test.
