# Supplementary material for: SARS-CoV-2 triggers Dickkopf-1 (Dkk-1) modulation of T helper cells and lung pathology in mice
Source: Genes Dis. 2023 Nov 15;11(4):101167. doi: 10.1016/j.gendis.2023.101167 (PMC10865256; doi:10.1016/j.gendis.2023.101167)
Supplement: Multimedia component 2 [file mmc2.docx]

SARS-CoV-2 is a novel coronavirus that has caused significant morbidity and mortality worldwide during the COVID-19 pandemic. However, the immune mechanisms underlying how SARS-CoV-2 causes severe lung pathology remains largely unknown. Here we show that SARS-CoV-2 spike protein interacts with ACE-2 receptors on platelets to cause release of platelet-derived Dkk-1. Dkk-1 is a wnt signaling inhibitor that has previously been shown to modulate CD4^+^ T helper cell differentiation during pulmonary infection. We found that release of Dkk-1 during SARS-CoV-2 infection causes dysregulation of CD4^+^ T helper cells leading to increased Th2 and Th17 cells and decreased Th1 cells in the lungs.  Dysregulation of CD4^+^ T helper cells results in high SARS-CoV-2 viral titers and lung pathology. However, treatment with a Dkk-1 inhibitor modulates the CD4^+^ T helper cell response increasing Th1 cells and normalizing Th2 and Th17 cells which enhances anti-viral immunity while preventing lung pathology. Together, the data suggest Dkk-1 may be an important molecule to target for future drug development against coronaviruses. (Illustrative figure generated at biorender.com)
